# Correction to Prevalence of type I Gaucher disease in patients with smoldering or multiple myeloma: Results from the prospective, observational CHAGAL study

**DOI:** 10.1002/hem3.70142

**Published:** 2025-05-14

Morè S, Federici I, Bossi A, et al. Prevalence of type I Gaucher disease in patients with smoldering or multiple myeloma: results from the prospective, observational CHAGAL study. *HemaSphere*. 2025;9:e70079. https://doi.org/10.1002/hem3.70079

In the author listing of the manuscript, the name of an author was incorrectly listed as Tommaso C. De Toritto. The correct name is Tommaso Caravita di Toritto.

Additionally, the author listing requires further amendment due to an error in the initial manuscript draft. As such, Angela Rago is now included as a co‐author.

The original article has been updated. We apologize for this error.

